# In Vitro Effects of Minor Olive Oil Compounds on Prostacyclin/Thromboxane Balance Under Acute High-Glucose Conditions

**DOI:** 10.3390/biom16050666

**Published:** 2026-04-30

**Authors:** Ana María Sánchez-Tévar, Laura Ortega-Hombrados, María Dolores Rodríguez-Pérez, María Monsalud Arrebola-Ramírez, Esther Martín-Aurioles, Sergio Pérez-Burillo, Cristina Verdugo-Cabello, Rocío Cobos-López, José Pedro De La Cruz, José Antonio González-Correa

**Affiliations:** 1 Departamento de Farmacología, Facultad de Medicina, Universidad de Málaga, IBIMA Plataforma BIONAND, Instituto de Investigación Biomédica de Málaga, 29010 Málaga, Spain; amstevar@uma.es (A.M.S.-T.); loladoct@uma.es (M.D.R.-P.); spburillo@uma.es (S.P.-B.); cristinaverdugocabello@uma.es (C.V.-C.); rociocl@uma.es (R.C.-L.); jpcruz@uma.es (J.P.D.L.C.); correa@uma.es (J.A.G.-C.); 2 UGC Laboratorio Clínico, Hospital de la Axarquía, Área de Gestión Sanitaria Este de Málaga-Axarquía, 29740 Málaga, Spain; mariam.arrebola.sspa@juntadeandalucia.es; 3 Distrito Sanitario Málaga-Guadalhorce, UGC La Roca, 29009 Málaga, Spain; estherd.martin.sspa@juntadeandalucia.es

**Keywords:** thromboxane, prostacyclin, oxidative stress, phenolic compounds, triterpenoids

## Abstract

This study aimed to evaluate the effects of several minor components of extra virgin olive oil (EVOO) on platelet thromboxane and vascular prostacyclin production in rat aortic rings under high-glucose conditions (300 mg/dL), in relation to their potential antioxidant actions. Under hyperglycaemic conditions, thromboxane production was 1.3 times higher, while prostacyclin production was 40.9% lower than in samples with 100 mg/dL glucose in aortic rings, accompanied by marked oxidative stress (65.6% higher than in samples with 100 mg/dL glucose). The compounds tested inhibited thromboxane production in a concentration-dependent manner, with relative potencies (secoiridoid derivatives (IC_50_ range: 10^−6^ M) = triterpenes (10^−6^ M) > alcoholic phenols (10^−5^ M for hydroxytyrosol and 10^−4^ M for the rest)), while preserving prostacyclin production (5–20% inhibition). All compounds also exerted vascular antioxidant effects, reducing oxidative stress markers and enhancing antioxidant parameters (IC_50_ range: 10^−6^–10^−5^ M), and these effects were observed under both normoglycaemic (100 mg/dL) and hyperglycaemic (300 mg/dL) conditions.

## 1. Introduction

It is well known that maintaining vascular prostacyclin (PGI_2_) synthesis is important for antithrombotic function [1], and vascular prostacyclin acts as a vasodilator and antiplatelet agent, playing a key role in the pathophysiology of ischemic cardiovascular disease [2]. In contrast, platelet-derived thromboxane A_2_ (TxA_2_) is a vasoconstrictor and pro-aggregatory prostanoid involved in thrombosis [2]. Both compounds are synthesized from arachidonic acid, and the balance between them (the “prostanoid balance”) is crucial for haemostasis or, conversely, for the development of a thrombotic event (e.g., reduced prostacyclin and/or increased thromboxane A_2_) [3].

Vascular prostacyclin has a very short half-life, and oxidative stress is a major factor in its degradation and inactivation [4]. Therefore, under conditions of excessive free radical production, such as in diabetes mellitus, its production is diminished, contributing to diabetic vasculopathy [5]. In addition, hyperglycaemia increases platelet thromboxane A_2_ production [6].

Since oxidative stress degrades prostacyclin, antioxidants have been proposed to increase its half-life and concentration [7]. Extra virgin olive oil (EVOO), the main fat source in the Mediterranean diet, is known for its cardiovascular benefits [8], and its minor components (phenols and triterpenes) are believed to contribute significantly to these protective effects [9]. Some of these compounds, such as hydroxytyrosol or 3,4-dihydroxyphenylglycol (DHPG), have shown protective effects against diabetic vasculopathy [10,11,12]. However, no studies have directly compared these effects among the different classes of minor EVOO components.

Therefore, this study aimed to compare the in vitro effects of the main minor EVOO components on the thromboxane A_2_–prostacyclin balance and to assess their antioxidant effects in rat aortic tissue under normoglycaemic (100 mg/dL) and hyperglycaemic (300 mg/dL) conditions.

## 2. Materials and Methods

### 2.1. Materials

The thiobarbituric acid-reactive substances (TBARS) colorimetric and 3-nitrotyrosine (3-Nty) enzyme immunoassay kits were obtained from Cell Biolabs Inc. (Bionova Científica S.L., Madrid, Spain), while the thromboxane B_2_ (TxB_2_) and 6-keto-prostaglandin F_1α_ (6-keto-PGF_1α_) enzyme immunoassay kits were obtained from Cayman Chemical (Ann Arbor, MI, USA). Lastly, the glutathione assay kits (for reduced-GSH and oxidized-GSSG- glutathione) and the glutathione peroxidase (GSHpx) activity kit, as well as 8-hydroxy-2′-deoxyguanosine (8-OHdG) assay kits, were obtained from Abcam plc (Cambridge, UK), and all other reagents were from Merck Life Science S.L.U. (Madrid, Spain).

Similarly, the minor EVOO components used in this study were supplied by Merck Life Science S.L.U. (Madrid, Spain) and included the following groups:

− Secoiridoid phenolic compounds: oleacein and oleocanthal.
− Alcoholic phenolic compounds: hydroxytyrosol, tyrosol, and 3,4-dihydroxyphenylglycol (DHPG).
− Triterpenic compounds: oleanolic acid and maslinic acid.

### 2.2. Study Protocol and Ethics

This experimental study used both male and female Wistar rats (200–250 g) housed at the University of Málaga Experimental Centre (CECA). After a one-week acclimation period, the animals were individually identified and housed in individual cages until study completion. Food and water were provided ad libitum, and daily consumption was recorded. The study conformed to the Declaration of Helsinki and Spanish laws (Law 14/2007 on Biomedical Research), and experimental protocols were approved by the Ethics Committee of the University of Málaga (Ref. CEUMA31-2018-A) and the Andalusian government (Ref. 9/07/2019/124). Additionally, all procedures followed Spanish regulations for animal care and use (Real Decreto Ley 2013/80847 and Boletin Oficial del Estado-A-2013-6271) and the NIH Guide for the Care and Use of Laboratory Animals.

### 2.3. Samples

A total of 84 Wistar rats (42 males and 42 females) were used. After the adaptation period, animals were anesthetized with sodium pentobarbital (40 mg/kg i.p.), and a blood sample was obtained by puncturing the iliac artery bifurcation and anticoagulated with 3.8% sodium citrate (1:10 *v*/*v*). The animals were then perfused with isotonic saline (pH 7.4) and decapitated. The entire aortic artery was excised, from the thoracic aorta to 1 cm below the renal artery bifurcation. It was then carefully dissected, and 3 mm aortic rings were prepared and incubated in isotonic saline until use.

### 2.4. Experimental Design

In this in vitro study, two factors were combined in each experiment:

− Glucose concentration: normoglycaemic (100 mg/dL; 5.5 mmol/L) or hyperglycaemic (300 mg/dL; 16.65 mmol/L), reflecting physiological and diabetic conditions, respectively.
− Compound concentration: Each sample was incubated with increasing concentrations of one test compound.

Experiments were conducted as follows:

− Aortic rings: Each ring was incubated in a buffer (mmol/L: NaCl: 100, KCl: 4, NaHCO_3_: 25, Na_2_SO_4_: 2.1, sodium citrate: 20, and Tris: 50; pH 7.4) containing glucose until a concentration of either 100 mg/dL or 300 mg/dL was reached. The rings were pre-incubated at 37 °C for 10 min in an oxygenated environment (95% O_2_; 5% CO_2_), and test compounds (at various concentrations) were then added and incubated for an additional 10 min at 37 °C. Prostacyclin synthesis was induced by adding 1 µM calcium ionophore (A23187). After 20 min, the samples were centrifuged (3000× *g*, 4 °C, 5 min), and aliquots of the supernatant were frozen at −80 °C for later determination of 6-keto-PGF1α (a stable prostacyclin metabolite). The aortic rings were then cold-homogenized (1:100 *w*/*v*) in a 0.05% protease inhibitor buffer (50 mM Tris-HCl, 150 mM NaCl, 1% Igepal CA-630, 0.5% sodium deoxycholate, and 0.1% SDS). The homogenates were sonicated and centrifuged (3000× *g*, 4 °C, 20 min), and the supernatants were frozen at −80 °C until biochemical analyses.
− Blood samples: Basal glucose was measured in each blood sample using a glucometer (FreeStyle, Abbott, Madrid, Spain) to determine the glucose volume needed to achieve 100 or 300 mg/dL. If basal glucose concentrations exceeded 100 mg/dL, the sample was only used for the 300 mg/dL condition. Each blood sample was incubated at 37 °C for 10 min in an oxygenated environment (95% O_2_, 5% CO_2_), and test compounds (various concentrations) were then added and incubated for 10 min. Thromboxane A_2_ synthesis was induced by 1 µM calcium ionophore (A23187). After 20 min, the samples were centrifuged (3000× *g*, 4 °C, 5 min), and aliquots of the supernatant were frozen at −80 °C for later determination of thromboxane B_2_ (the stable metabolite of thromboxane A_2_).

### 2.5. Analytical Determinations

#### 2.5.1. Blood Samples

Thromboxane B_2_ expression in the induced blood sample supernatants was quantified by enzyme immunoassay following the manufacturer’s instructions.

#### 2.5.2. Aortic Ring Samples

The following determinations were performed on the aortic ring homogenates or supernatants:

− Lipid peroxidation (TBARS): Thiobarbituric acid-reactive substances (TBARS) were measured to estimate malondialdehyde (MDA) levels as an index of lipid peroxidation. Briefly, 100 μL of sample or MDA standards was mixed with 250 μL of 5.2 mg/mL thiobarbituric acid (pH 3.5). Samples were then incubated at 95 °C for 45 min and centrifuged at 3000× *g* for 15 min. Supernatant absorbance was measured at 532 nm, and MDA concentrations were calculated from a standard curve.
− 8-Hydroxy-2′-deoxyguanosine (8-OHdG): 8-OHdG concentration in aortic tissue was determined as a marker of oxidative DNA damage using an enzyme immunoassay kit according to the manufacturer’s protocol.
− 3-Nitrotyrosine: 3-Nitrotyrosine expression was measured as an index of peroxynitrite formation, and samples were assayed by enzyme immunoassay following the kit’s instructions. Briefly, 50 μL of the sample or standard was incubated with 50 μL of the anti-3-nitrotyrosine antibody for 1 h at room temperature. After washing, 100 μL of the secondary antibody was added for 1 h, followed by substrate addition and absorbance reading at 450 nm. Concentrations were determined from a standard curve.
− Reduced (GSH) and oxidized (GSSG) glutathione: Aortic rings were homogenized in 50 mM phosphate-buffered saline (pH 7.0) containing 5% sulfosalicylic acid (1:25 *w*/*v*). Homogenates were centrifuged (8000× *g*, 10 min, 4 °C), and supernatants were frozen until analysis. Total GSH and GSSG were measured using a recycling assay (Ellman’s reagent and glutathione reductase). For GSH, samples were assayed directly, and for GSSG, samples were first treated with 4-vinylpyridine to derivatize GSH before the assay. The percentage of GSSG was calculated as %GSSG = (GSSG/(GSH + GSSG)) × 100.
− Glutathione peroxidase (GSHpx) activity: GPx activity was measured using a colorimetric kit based on NADPH oxidation at 340 nm, as GSH is converted to GSSG.
− 6-keto-PGF1α: Aortic ring supernatants (collected before homogenization) were assayed for 6-keto-PGF1α, the stable metabolite of prostacyclin, using an enzyme immunoassay kit according to the manufacturer’s instructions.

### 2.6. Statistical Analysis

Data are expressed as the mean ± standard deviation (SD; 6 samples per condition). For each variable, the effect of compound incubation was expressed as a percentage of the baseline (no compound) value: %post-incubation = (post-incubation value/baseline value) × 100. These percentages were used to plot concentration–effect curves, allowing the IC_50_ (the concentration that inhibits 50% of the baseline effect) to be determined. For this purpose, an Excel spreadsheet was used, with one column containing the concentrations of the compound used (*X*-axis) and another containing the percentages of the effect obtained with each concentration relative to the values of the control samples (*Y*-axis); the X values were transformed into logarithms, and a linear regression of the form Y = aX + b was obtained, with the value of X determined using Y = 50; the antilogarithm of the value obtained was calculated, and this was the IC_50_ value.

The number of animals used was calculated based on previous experiments conducted by our research group while considering two parameters: TBARS production as an indicator of antioxidant activity and thromboxane B2 production as an indicator of a possible effect on prostanoid production. The following conditions were used when determining the optimal level: a specific reference compound (hydroxytyrosol) should exceed 50% inhibition, and any other compound should not exceed 50% inhibition. With a 95% confidence level, a statistical power of 0.80, and a 10% variability, as estimated in previous studies, the sample size was calculated as 4.21 per group. Since some loss of cases during the course of the experiments was expected, it was decided that six rats per group were needed, thereby establishing the replicates at the biological level.

Statistical comparisons were performed using SPSS v.25 (IBM, Chicago, IL, USA), and an unpaired Student’s *t*-test was used to compare the means of the basal values in the glucose 100 mg/dL samples with respect to basal values in the glucose 300 mg/dL samples. In addition, a two-way ANOVA was performed with the following factors: glucose concentration and IC_50_ values of the various compounds used. Tukey’s post hoc test was applied, and *p* < 0.05 was considered statistically significant.

## 3. Results

No statistically significant differences were observed between the values obtained from male and female animals; therefore, to clarify the results, each result described represents the mean of a pool of samples from male and female rats in each group.

### 3.1. Effect of Glucose Concentration

All measured variables (prostanoid balance and oxidative stress markers) were significantly altered in aortic rings incubated with 300 mg/dL glucose compared to those under the 100 mg/dL condition (Table 1). Under hyperglycaemic conditions, oxidative stress markers increased by 65.6% with respect to the glucose 100 mg/dL group (96.8% for TBARS, 57.4% for 8-OHdG, and 42.8% for 3-nitrotyrosine), while antioxidant markers were modified by 122% (46.6% for GSH, 121% for GSSG, and 150% for GSHpx). Platelet thromboxane production was 1.3 times higher in samples with a glucose concentration of 300 mg/dL, while aortic prostacyclin production was reduced by 40.9% in samples with a glucose concentration of 300 mg/dL; the thromboxane/prostacyclin ratio was 1.21 ± 0.17 at 100 mg/dL glucose and 4.76 ± 0.25 at 300 mg/dL (2.9 times higher, *p* < 0.05).

### 3.2. Effect of Compounds on Prostanoid Balance

**Figure 1:**
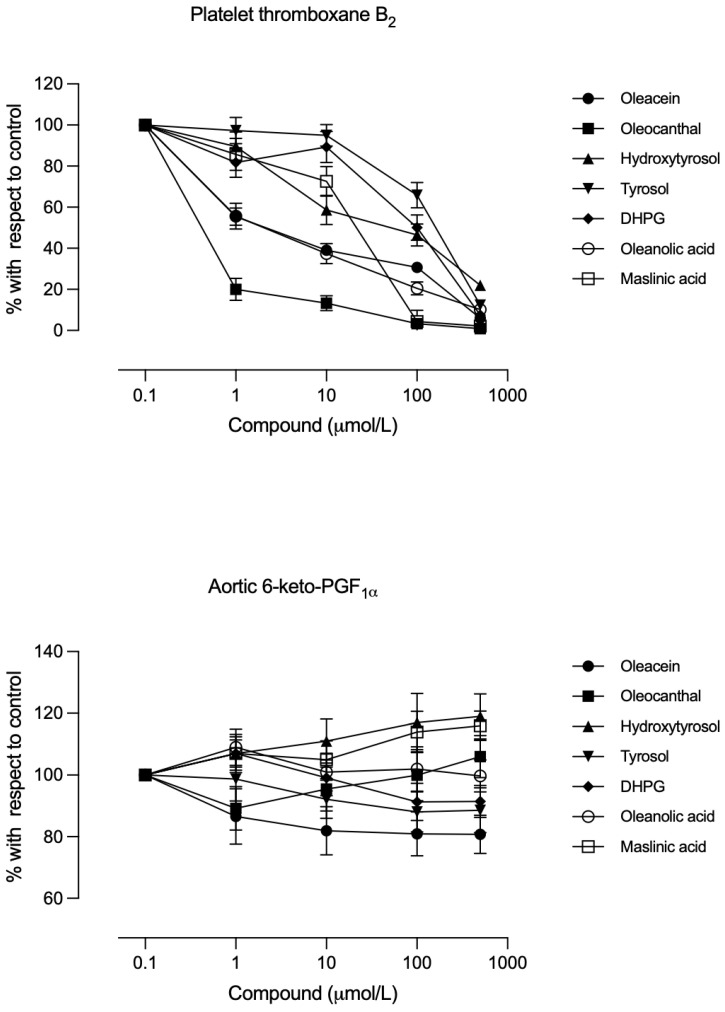
Concentration–effect curves of the studied compounds on platelet thromboxane B_2_ (**upper panel**) and aortic ring 6-keto-PGF_1α_ (**bottom panel**) levels after incubation with 300 mg/dL glucose. Each point is the mean ± standard deviation of 5 aortic rings or blood samples from each of the 6 different animals per group; each group corresponds to a different compound.

All tested compounds decreased platelet thromboxane B_2_ production in a concentration-dependent manner. Figure 1 (upper panel) shows the dose–response curves under the 300 mg/dL glucose condition, and the IC_50_ values for each compound are given in Table 2 (glucose 100 mg/dL) and Table 3 (glucose 300 mg/dL). At both 100 and 300 mg/dL glucose concentrations, the potency order for inhibiting thromboxane B_2_ production was as follows: secoiridoid phenols (10^−6^ M) = triterpenes (10^−6^ M) > alcoholic phenols (10^−4^ M). No significant differences in IC_50_ values were observed between the two glucose levels (*p* > 0.05). Table 4 shows the statistical analysis indicating the differences in the IC_50_ values of the compounds in inhibiting thromboxane B_2_ production.

The production of 6-keto-PGF_1α_ (a prostacyclin metabolite) was not inhibited below 50% by any compound at either glucose level (Figure 1, lower panel), and the maximum effects observed ranged from a reduction of 5–18% (for tyrosol, oleacein, and DHPG) to an increase of 5–20% (for oleocanthal, maslinic acid, and hydroxytyrosol) relative to baseline. Oleanolic acid did not significantly alter 6-keto-PGF1α levels compared to baseline.

### 3.3. Effect of Compounds on Vascular Oxidative Markers

**Figure 2:**
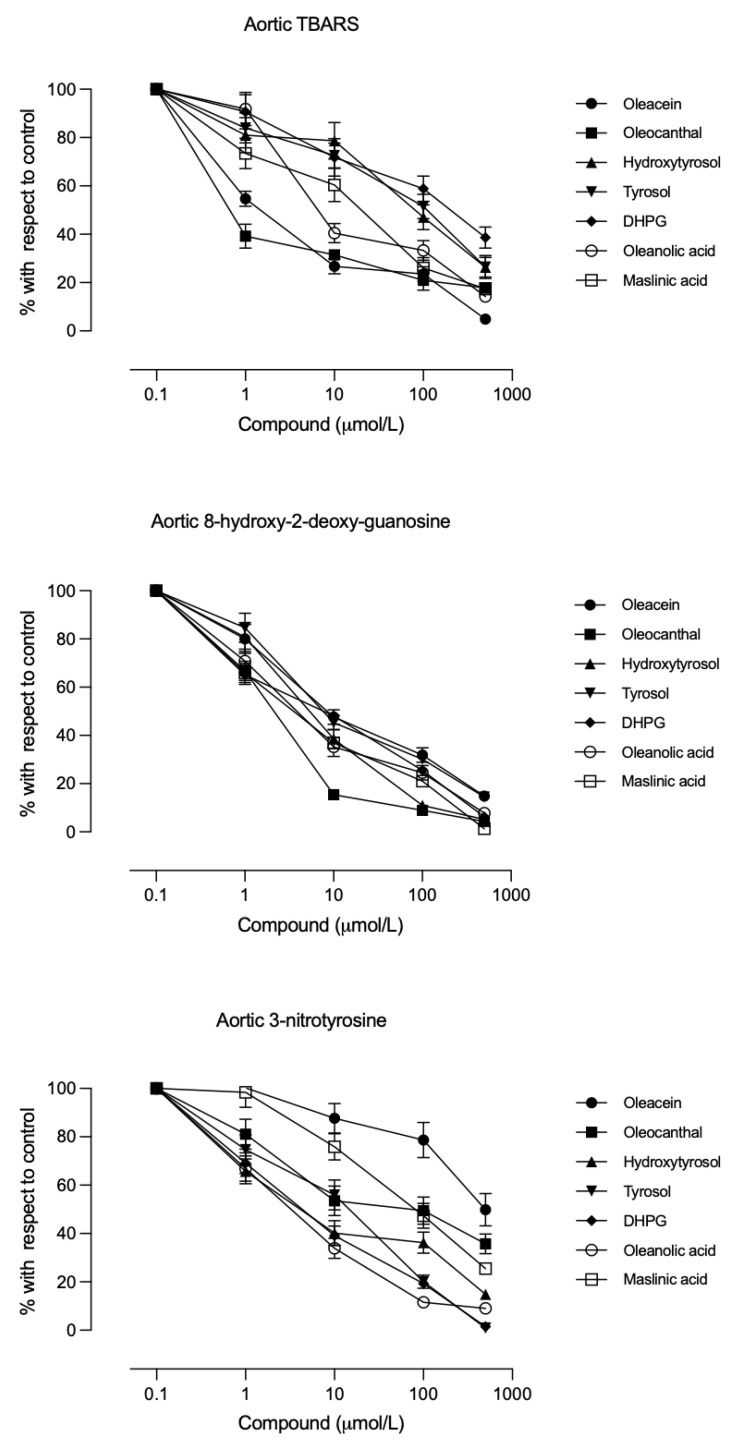
Concentration–effect curves of the studied compounds on the aortic rings thiobarbituric acid-reactive substances (TBARS) (**upper panel**), 8-hydroxy-2-deoxy-guanosine (**medium panel**), and 3-nitrotyrosine (**bottom panel**) levels after incubation with 300 mg/dL glucose. Each point is the mean ± standard deviation of 5 aortic rings from each of the 6 different animals per group; each group corresponds to a different compound.

All compounds inhibited TBARS, 8-OHdG, and 3-nitrotyrosine production in a concentration-dependent manner at both glucose concentrations. Figure 2 shows the response curves under the 300 mg/dL glucose condition, and the IC_50_ values for each compound are shown in Table 2 (glucose 100 mg/dL) and 3 (glucose 300 mg/dL). The order of potency (at either glucose level) was as follows: secoiridoid phenols (10^−6^ M) > triterpenes (10^−6^–10^−5^ M) > alcoholic phenols (10^−4^ M). No significant differences were found between the two glucose conditions. Table 5 shows the statistical analysis indicating the differences in the IC_50_ values of the compounds in inhibiting the aortic rings oxidative markers.

### 3.4. Effect of Compounds on Vascular Antioxidant Markers

**Figure 3:**
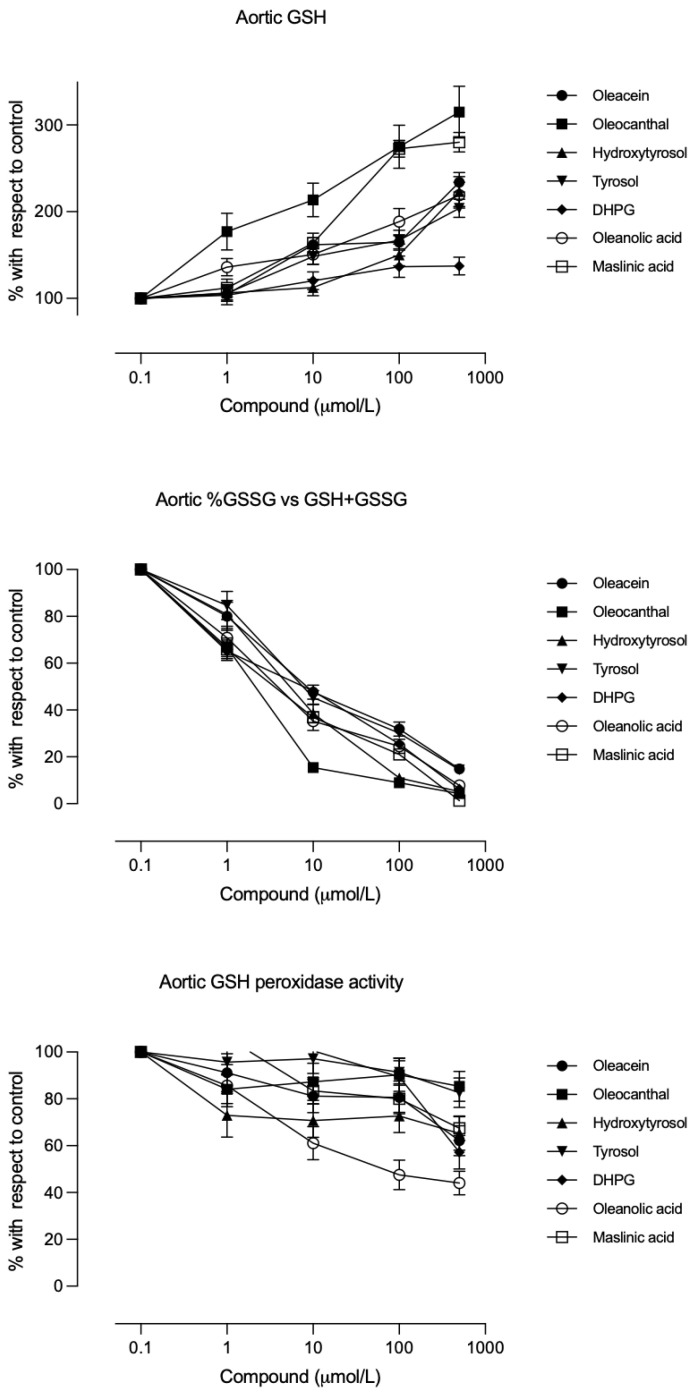
Concentration–effect curves of the studied compounds on the aortic rings reduced glutathione (GSH) level (**upper panel**), percentage of oxidized glutathione (GSSG) with respect to total glutathione concentration (**medium panel**), and glutathione peroxidase activity (GSHpx) (**bottom panel**) after incubation with 300 mg/dL glucose. Each point is the mean ± standard deviation of 5 aortic rings from each of the 6 different animals per group; each group corresponds to a different compound.

All compounds increased GSH levels in a concentration-dependent manner under both normoglycaemic and hyperglycaemic conditions (Figure 3, upper panel). The maximal increases observed ranged from 27% (for DHPG) up to 2.8–3.1-fold above baseline for oleocanthal and maslinic acids and 2.0–2.4-fold for the other compounds. No difference between glucose conditions was noted for this effect.

Glutathione peroxidase (GSHpx) activity (Figure 3, lower panel) and the percentage of glutathione in its oxidized form (GSSG%, Figure 3, middle panel) were inhibited by all compounds in a concentration-dependent manner. Only oleanolic acid achieved 50% inhibition of GSHpx at 300 mg/dL glucose (IC_50_ ≈ 500 µM; Table 3), and all compounds showed similar potency for reducing GSSG% at both glucose levels (Table 2 and Table 3). Table 6 shows the statistical analysis indicating the differences in the IC_50_ values of the compounds in modifying the aortic rings antioxidant markers.

## 4. Discussion

This study makes two key contributions to the literature. First, it evaluates, within the same experimental platform, three major classes of minor extra virgin olive oil (EVOO) constituents—secoiridoids, alcoholic phenols, and triterpenes—under identical normoglycaemic and hyperglycaemic conditions. Second, it links their effects on prostanoid production to a broad panel of oxidative and nitrosative stress markers in vascular tissue. Taken together, the data support a coherent interpretation: acute high-glucose exposure induces a prothrombotic redox phenotype characterized by enhanced thromboxane generation, reduced prostacyclin bioavailability, lipid and DNA oxidation, protein nitration, depletion of reduced glutathione, and oxidation of the glutathione pool. Against this background, the tested EVOO compounds consistently reduced thromboxane B_2_ formation, largely preserved 6-keto-PGF_1α_, and improved multiple oxidative stress endpoints. This pattern is pharmacologically attractive because it points to a selective correction of the thromboxane/prostacyclin disequilibrium rather than a non-specific suppression of prostanoid synthesis.

Our results provide a comprehensive overview of the effects of the main groups of minor EVOO compounds on vascular and platelet prostanoid production, as well as their known antioxidant actions in rat arterial tissue [13]. Importantly, these effects were observed under hyperglycaemic conditions relevant to diabetic cardiovascular risk.

Hyperglycaemia is a major risk factor for cardiovascular disease and initiates pathways leading to diabetic macro- and microangiopathy [14]. These pathways (polyol, advanced glycation, protein kinase C, and hexosamine) all generate oxidative stress, which in turn exacerbates them [15]. In our aortic model, hyperglycaemia induced a state of vascular oxidative stress, as evidenced by increased oxidative marker expression and decreased antioxidant defenses, as well as nitrosative stress (Table 1)—characteristics essentially describing a complete oxidative stress state. Concurrently, a thromboxane/prostacyclin imbalance was observed (Table 1), with increased platelet thromboxane production and decreased prostacyclin production. This imbalance is also an important factor in the development of diabetic vasculopathy and its ischemic–thrombotic complications [5].

The present data allow strong statements about the phenotypic output—less thromboxane, preserved prostacyclin, and lower oxidative damage—but they do not identify the proximal molecular targets. No direct measurements of COX-1, COX-2, thromboxane synthase, prostacyclin synthase, phospholipase A_2_, eNOS, NADPH oxidases, mitochondrial ROS, Nrf2 signaling, or inflammatory transcription factors were made. For that reason, it would be excessive to attribute the observed effects to a specific cyclooxygenase isoform or to claim definitive preservation of prostacyclin synthase activity. Likewise, because thromboxane expression was measured after calcium ionophore stimulation in whole blood rather than in isolated platelets, the assay captures platelet-dependent thromboxane generation but does not completely dissect platelet-intrinsic signaling from the possible influences of other blood components. Future studies should address these mechanistic gaps by combining enzyme expression/activity analyses, endothelial and platelet cell models, pharmacological antagonism, and perhaps untargeted lipidomics to define how the eicosanoid network is being remodeled.

All tested EVOO compounds inhibited platelet thromboxane production, suggesting a possible inhibition of cyclooxygenase-1 (COX-1) activity in platelets. This effect was more pronounced for secoiridoid and triterpenic derivatives than for alcoholic phenols (Table 2 and Table 3), and such COX inhibition and anti-inflammatory activity by these compounds are well documented (for example, secoiridoid derivatives [16,17,18], triterpenes [10,19,20,21], and alcoholic phenols [22,23,24]). Systemically, COX inhibition implies potential anti-inflammatory effects, which are important in the cardiovascular system, as COX-derived prostanoids are critical for platelet–vessel interactions and blood flow. Indeed, animal studies show that certain EVOO phenolics (alone or combined with triterpenes) reduce vascular lesions in diabetes mellitus [11,19,25]. Our findings suggest that these benefits may arise in part from reducing the hyperglycaemia-induced prostanoid imbalance.

Notably, the compounds had a selective effect: platelet thromboxane production was strongly inhibited, but vascular prostacyclin production was largely spared (Figure 1). At concentrations causing 50% TXA_2_ inhibition, prostacyclin was only inhibited modestly (e.g., ~7–16% for oleacein and oleocanthal, with some compounds even showing slight stimulation). In other words, the inhibitory effect on platelet TXA_2_ was much greater than on vascular PGI_2_. This is compatible with biosynthetic pathways: platelet TXA_2_ is COX-1-dependent [26], whereas vascular PGI_2_ is largely COX-2-dependent, especially under shear stress or vascular inflammation [27,28]. Some reports suggest that EVOO compounds inhibit COX-1 and COX-2 similarly [17], while others find greater COX-2 inhibition [29,30]. Regardless, our data indicate a differential effect: platelet TXA_2_ is inhibited, while vascular PGI_2_ is preserved. This implies a mechanism protecting prostacyclin synthesis. By contrast, nonselective COX inhibitors like aspirin reduce both TXA_2_ and PGI_2_ expression [28]. In rats, oral hydroxytyrosol similarly reduced platelet TXA_2_ expression more than that of PGI_2_ [24,31]. One possible explanation is the antioxidant capacity of these phenolics, which can protect prostacyclin synthase from inactivation by reactive oxygen species under high-glucose conditions [32].

From a pharmacological perspective, this is indicative of a more favorable profile than a non-selective depression of both pathways. It suggests that the studied compounds do not simply behave as indiscriminate cyclooxygenase inhibitors, or at least that any direct inhibitory action on the cyclooxygenase pathway is counterbalanced by the simultaneous preservation of endothelial prostacyclin-generating capacity. This distinction matters because the clinical utility of antiplatelet or anti-inflammatory interventions in cardiometabolic disease is heavily influenced by whether they preserve vasoprotective eicosanoid signaling [27]. A recent translational study also highlights that endothelial-derived prostanoids actively modulate platelet behavior in diabetes; for example, hyperglycaemia-driven endothelial reprogramming can favor platelet activation through altered eicosanoid production, underscoring the importance of the vascular compartment in the diabetic prothrombotic state. Thus, 6-keto-PGF_1α_ preservation in the present study may be as mechanistically relevant as the decrease in thromboxane B_2_ expression.

The most parsimonious explanation is that antioxidant and antinitrosative effects contribute substantially to this prostacyclin-sparing profile. Prostacyclin synthase is known to be vulnerable to oxidative and nitrative inactivation, while endothelial NO depletion and excess ROS further shift the vascular wall towards a dysfunctional, pro-aggregatory phenotype [14]. In the current experiments, all compound classes reduced markers that reflect lipid peroxidation, oxidative DNA injury, and protein nitration. The reduction in 3-nitrotyrosine is especially noteworthy because nitrative stress is mechanistically linked to endothelial enzyme dysfunction and the loss of vasoprotective signaling. Therefore, one plausible interpretation is that these compounds largely preserve the functional integrity of the prostacyclin axis by preventing its oxidative suppression rather than by stimulating its synthesis directly. This is, however, an inference rather than a direct demonstration, and it should be presented as such. Neither prostacyclin synthase activity nor cyclooxygenase isoform expression was measured. Consequently, the present data support a redox-associated preservation of prostacyclin bioavailability, but they do not yet establish the molecular target responsible for it. The inhibition of COX activity in platelets and the vascular wall may not be the only mechanism explaining the effect observed with the compounds used, as other pathways that could account for this effect, either on their own or in addition to COX inhibition, should be considered (for example, differential uptake, local redox effects on prostacyclin synthase, and differences in the compartmentalization of prostanoid synthesis).

In this study, all minor EVOO compounds produced antioxidant effects in aortic tissue under both normoglycaemic and hyperglycaemic conditions (Figure 2 and Figure 3). The antioxidant properties of these phenols and triterpenes are well documented [13,33,34]. Under high-glucose conditions, such antioxidant action could prevent the hyperglycaemia-induced decrease in prostacyclin [4,7], thereby preserving the vasodilatory, antiplatelet mediator (prostacyclin) while inhibiting platelet TXA_2_ synthesis.

A particularly relevant point in the interpretation of these results is the translation from isolated compounds to the real nutritional matrix of EVOO. EVOO is not a simple sum of independent molecules; it is a chemically complex system in which minor constituents may act additively or synergistically and where bioavailability is influenced by the lipid environment [9,34]. The recent clinical and translational literature continues to support the view that higher-phenolic EVOO preparations can improve endothelial function, haemodynamic parameters, oxidative stress markers, and inflammatory profiles, especially in cardiometabolic risk populations [35,36]. In addition, hydroxytyrosol-centered intervention studies suggest measurable changes in platelet-related pathways and redox metabolism in humans [37]. These data strengthen the translational plausibility of the present findings, but they also highlight a key limitation: the effects of purified compounds tested acutely in vitro cannot be assumed to reproduce the effects of dietary EVOO when consumed chronically. The EVOO matrix contains monounsaturated fatty acids, minor lipids, sterols, tocopherols, and multiple phenolic derivatives whose interactions may modify both pharmacodynamics and pharmacokinetics. Accordingly, the current data should be framed as mechanistic support for a plausible mode of action of minor EVOO constituents, not as direct evidence that isolated compounds will reproduce the cardiovascular effects of the whole oil in humans.

Two general points emerge from our results. First, the effects were seen at both physiological (100 mg/dL) and high (300 mg/dL) glucose levels, suggesting potential preventive benefits even under normoglycaemic conditions. This possibility would require primary prevention studies; however, EVOO’s antioxidant effects have already been demonstrated in healthy volunteers [37] and in cardiovascular risk patients [38]. Second, although all compounds were active, their potencies differed by endpoint: secoiridoid derivatives and triterpenes had stronger effects on prostanoid production and oxidative stress (Table 2 and Table 3). However, since all these compounds are present together in EVOO, synergistic interactions may occur. For example, combinations of oleocanthal with hydroxytyrosol or DHPG with hydroxytyrosol have shown synergistic antioxidant effects in rat brains [39]. It would be valuable to investigate such interactions in aortic tissue for both antioxidant actions and prostanoid production, and studies are underway in our laboratory.

A limitation concerns concentration and bioavailability. In vitro efficacy does not necessarily imply dietary attainability of the parent compounds in their free forms. Olive phenolics undergo extensive first-pass and phase II metabolism, while triterpenes show formulation-dependent and often limited oral bioavailability [9,33,34]. Accordingly, the micromolar concentrations that effectively suppress thromboxane or oxidative damage in this assay may not map directly onto circulating concentrations after normal dietary EVOO intake. This does not invalidate the mechanistic results, but it does affect translational interpretation. The most defensible wording is that this study identifies the pharmacodynamic capabilities of minor EVOO components under controlled experimental conditions and provides mechanistic support for the cardiovascular relevance of EVOO bioactives. It does not yet establish that the same isolated compounds, at nutritionally achievable exposures, will reproduce these quantitative effects in vivo. Bridging studies should therefore integrate pharmacokinetic measurements, metabolite testing, chronic dietary interventions, and disease models that more closely resemble type 2 diabetes.

The findings should nevertheless be discussed with appropriate caution because several limitations constrain mechanistic depth and translational reach. The model is acute and in vitro. Acute exposure of blood and aortic rings to 300 mg/dL glucose is useful to isolate the direct effects of excess glucose, but it cannot replicate the chronic metabolic, inflammatory, endocrine, and lipotoxic environment of diabetes. Endothelial dysfunction in diabetes develops over time and is shaped by insulin resistance, advanced glycation, circulating cytokines, altered lipoproteins, disturbed flow, mitochondrial remodeling, and epigenetic changes [5,14]. Therefore, the present model captures an important but reduced fragment of diabetic vasculopathy.

These results complement those obtained in previous studies by our group using an experimental model of diabetes mellitus [10,11,12,19,24,25]. In those studies, chronic hyperglycaemia led to oxidative stress and a prostanoid imbalance that is qualitatively similar to that found in this study in samples exposed to an acute glucose concentration of 300 mg/dL; likewise, the administration of hydroxytyrosol, DHPG, their combination, or an olive oil rich in triterpenes produced an antioxidant effect and a rebalancing of the thromboxane/prostacyclin ratio, demonstrating protection against vascular morphological damage. This study suggests, albeit at a very preliminary stage, that other minor components of EVOO exert a vascular effect, pointing to a potential preventive role in diabetes mellitus, whether administered alone or in combination, with a particular focus on secoiridoids and triterpenes, which have demonstrated a pronounced vascular effect.

Future research should proceed in several directions. First, the most active compounds—particularly oleacein, oleocanthal, oleanolic acid, and maslinic acid—should be studied in chronic diabetic models with integrated vascular, platelet, and metabolic phenotyping. Secondly, mechanistic studies should determine whether the prostacyclin-sparing effect reflects preservation of prostacyclin synthase, differential modulation of cyclooxygenase isoforms, protection of NO signaling, attenuation of NADPH oxidase activity, or a combination of these processes. Thirdly, combinations of compounds merit explicit study because the whole oil context strongly suggests synergy rather than isolated action [9,10,12,34,39]. Finally, translational studies should aim to connect circulating phenolic and triterpenic metabolites with biomarkers of platelet activation, endothelial function, and vascular inflammation in individuals with prediabetes or diabetes. Such work would allow the field to move from descriptive cardioprotection associated with EVOO towards a more mechanistically grounded nutraceutical pharmacology.

## 5. Conclusions

In rat aortic rings, acute high-glucose conditions induce marked oxidative stress and an imbalance in platelet thromboxane A_2_ vs. vascular prostacyclin (increased TXA_2_ and decreased PGI_2_). Under these conditions, the main minor components of extra virgin olive oil exert antioxidant effects in the vascular tissue and reduce platelet prostanoid production while preserving vascular prostacyclin synthesis, likely due to their strong antioxidant actions, thereby supporting EVOO’s acute protective effects.

Although this study’s findings are preliminary, they point to a potential area of intervention in the prevention of ischaemic cardiovascular disease, primarily in incidence prevention, either by adjusting the composition of extra virgin olive oil to include the most active compounds in this regard or as an adjunct to the antithrombotic medication used in these patients.

## Figures and Tables

**Table 1:** Basal values (mean ± standard deviation) of quantified variables after glucose incubation. N = 6 aortic rings from 6 different animals.

	Glucose Concentration	
	100 mg/dL	300 mg/dL
TBARS (nmol/mg protein) 8-OH-2-dG (nmol/mg protein)	11.38 ± 0.21 4.15 ± 0.29	22.40 ± 1.08 * 6.59 ± 0.51 *
3-nitrotyrosine (nmol/mg protein)	2.86 ± 0.15	4.89 ± 0.30 *
GSH (µmol/mg protein)	7.95 ± 0.37	4.24 ± 0.31 *
GSSG (µmol/mg protein)	0.33 ± 0.03	0.74 ± 0.08 *
%GSSG with respect to GSH + GSSG	4.05 ± 0.38	14.86 ± 1.53 *
GSHpx (µmol/min/mg protein)	0.29 ± 0.02	0.73 ± 0.03 *
Thromboxane B_2_ (pmol/mg protein)	0.96 ± 0.07	2.41 ± 0.23 *
6-keto-PGF_1α_ (nmol/mg protein)	2.96 ± 0.25	1.34 ± 0.09 *

* *p* < 0.05 with respect to samples with a glucose concentration of 100 mg/dL. TBARS: thiobarbituric acid-reactive substances. GSH: reduced glutathione. GSSG: oxidized glutathione. GSHpx: glutathione peroxidase activity. PGF_1α_: prostaglandin F_1α_. 8-OH-dG: 8-OH-2-deoxyguanosine.

**Table 2:** Mean values (mean ± standard deviation) of the concentration of each compound that inhibited 50% of the maximal production of each variable (IC_50_). N = 5 aortic rings from each of the 6 different animals per group; each group corresponds to a different compound. Glucose concentration = 100 mg/dL.

IC_50_ (µmol/L)							
	Secoiridoids		Alcoholic Phenols			Triterpenes	
	Oleacein	Oleocanthal	Hydroxytyrosol	Tyrosol	DHPG	Oleanolic Acid	Maslinic Acid
TBARS 8-OH-2-dG	2.89 ± 0.09 46.11 ± 3.90	0.77 ± 0.06 31.65 ± 3.88	108 ± 7.42 99.21 ± 7.04	175 ± 10.27 156 ± 8.48	276 ± 15.75 97.34 ± 7.77	12.15 ± 1.45 4.09 ± 0.64	43.45 ± 3.16 14.47 ± 1.17
3-nitrotyrosine	>500	75.33 ± 4.80	3.71 ± 0.17	20.67 ± 0.04	3.69 ± 0.03	1.82 ± 0.02	56.25 ± 3.19
GSH *	>500	>500	>500	>500	>500	>500	>500
GSSG	75.15 ± 5.20	70.22 ± 5.67	10.12 ± 1.38	75.41 ± 4.20	32.70 ± 2.71	10.56 ± 1.07	9.97 ± 0.75
%GSSG with respect to GSH + GSSG	10.11 ± 1.01	5.43 ± 0.48	6.04 ± 0.41	9.44 ± 1.01	8.78 ± 0.99	5.37 ± 0.40	6.12 ± 0.38
GSHpx	>500	>500	>500	>500	>500	>500	>500
Thromboxane B_2_	4.05 ± 0.22	1.48 ± 0.13	98.14 ± 6.25	276 ± 12.24	128 ± 9.85	5.15 ± 0.37	3.53 ± 0.25
6-keto-PGF_1α_	>500	>500	>500	>500	>500	>500	>500

TBARS: thiobarbituric acid-reactive substances. GSH: reduced glutathione. GSSG: oxidized glutathione. GSHpx: glutathione peroxidase activity. PGF_1α_: prostaglandin F_1α_. DHPG: 3,4-dihydroxy-phenyl-glycol. 8-OH-dG: 8-OH-2-deoxyguanosine. * All compounds increased GSH values.

**Table 3:** Mean values (mean ± standard deviation) of the concentration of each compound that inhibited 50% of the maximal production of each variable (IC_50_). N = 5 aortic rings from each of the 6 different animals per group; each group corresponds to a different compound. Glucose concentration = 300 mg/dL.

IC_50_ (µmol/L)							
	Secoiridoids		Alcoholic Phenols			Triterpenes	
	Oleacein	Oleocanthal	Hydroxytyrosol	Tyrosol	DHPG	Oleanolic Acid	Maslinic Acid
TBARS 8-OH-2-dG	2.57 ± 0.31 51.25 ± 3.48	0.85 ± 0.07 28.23 ± 3.10	89.28 ± 5.47 93.61 ± 8.36	134 ± 9.75 189 ± 9.22	248 ± 17.25 81.90 ± 6.24	8.75 ± 0.94 2.77 ± 0.12	38.36 ± 2.12 9.23 ± 0.74
3-nitrotyrosine	486 ± 62.73	80.11 ± 6.23	6.64 ± 0.42	24.75 ± 2.17	6.69 ± 0.41	5.61 ± 0.27	85.02 ± 5.21
GSH *	>500	>500	>500	>500	>500	>500	>500
GSSG	88.70 ± 5.22	75.54 ± 4.37	7.46 ± 0.50	69.25 ± 4.36	26.44 ± 1.58	8.47 ± 0.53	8.20 ± 0.44
%GSSG with respect to GSH + GSSG	9.23 ± 0.49	3.98 ± 0.25	7.52 ± 0.43	8.91 ± 0.54	9.11 ± 0.03	6.20 ± 0.48	5.66 ± 0.36
GSHpx	>500	>500	>500	>500	>500	498 ± 38.11	>500
Thromboxane B_2_	3.75 ± 0.42	0.62 ± 0.03	72.87 ± 6.13	215 ± 23.37	109 ± 16.2	4.16 ± 0.51	4.01 ± 0.28
6-keto-PGF_1α_	>500	>500	>500	>500	>500	>500	>500

TBARS: thiobarbituric acid-reactive substances. GSH: reduced glutathione. GSSG: oxidized glutathione. GSHpx: glutathione peroxidase activity. PGF_1α_: prostaglandin F_1α_. DHPG: 3,4-dihydroxy-phenyl-glycol. 8-OH-dG: 8-OH-2-deoxyguanosine. * All compounds increased GSH values.

**Table 4:** Statistical analysis of the effect (IC_50_) of the compounds used on platelet thromboxane B_2_ production in samples with glucose levels of 100 and 300 mg/dL, as determined using a two-way ANOVA test along with Tukey’s post hoc test.

Glucose 100 mg/dL							
	Oleacein	Oleocanthal	HTy	Ty	DHPG	Oleanolic Acid	Maslinic Acid
Oleacein	-----	n.s.	<0.05	<0.05	<0.05	n.s.	n.s.
Oleocanthal	n.s.	-----	<0.05	<0.05	<0.05	n.s.	n.s.
HTy	<0.05	<0.05	-----	<0.05	<0.05	<0.05	<0.05
Ty	<0.05	<0.05	<0.05	-----	<0.05	<0.05	<0.05
DHPG	<0.05	<0.05	<0.05	<0.05	-----	<0.05	<0.05
Oleanolic acid	n.s.	n.s.	<0.05	<0.05	<0.05	-----	n.s.
Maslinic acid	n.s.	n.s.	<0.05	<0.05	<0.05	n.s.	-----
**Glucose 300 mg/dL**							
	**Oleacein**	**Oleocanthal**	**HTy**	**Ty**	**DHPG**	**Oleanolic Acid**	**Maslinic Acid**
Oleacein	-----	<0.05	<0.05	<0.05	<0.05	n.s.	n.s.
Oleocanthal	<0.05	-----	<0.05	<0.05	<0.05	<0.05	<0.05
HTy	<0.05	<0.05	-----	<0.05	<0.05	<0.05	<0.05
Ty	<0.05	<0.05	<0.05	-----	<0.05	<0.05	<0.05
DHPG	<0.05	<0.05	<0.05	<0.05	-----	<0.05	<0.05
Oleanolic acid	n.s.	<0.05	<0.05	<0.05	<0.05	-----	n.s.
Maslinic acid	n.s.	<0.05	<0.05	<0.05	<0.05	n.s.	-----

HTy: hydroxytyrosol. Ty: tyrosol. DHPG: 3,4-dihydroxyphenylglycol. n.s.: non-significant differences.

**Table 5:** Statistical analysis of the effect (IC_50_) of the compounds used on the aortic rings oxidative markers (TBARS, 8-OH-2-deoxyguanosine, and 3-nitrotyrosine) in samples with glucose levels of 100 and 300 mg/dL, as measured using a two-way ANOVA test along with Tukey’s post hoc test.

Glucose 100 mg/dL							
	Oleacein	Oleocanthal	HTy	Ty	DHPG	Oleanolic Acid	Maslinic Acid
Oleacein	-----	n.s.	<0.05	<0.05	<0.05	<0.05	<0.05
Oleocanthal	n.s.	-----	<0.05	<0.05	<0.05	<0.05	<0.05
HTy	<0.05	<0.05	-----	<0.05	<0.05	<0.05	<0.05
Ty	<0.05	<0.05	<0.05	-----	n.s.	<0.05	<0.05
DHPG	<0.05	<0.05	<0.05	<0.05	-----	<0.05	<0.05
Maslinic acid	<0.05	<0.05	<0.05	<0.05	<0.05	<0.05	-----
**Glucose 300 mg/dL**							
	**Oleacein**	**Oleocanthal**	**HTy**	**Ty**	**DHPG**	**Oleanolic Acid**	**Maslinic Acid**
Oleacein	-----	n.s.	<0.05	<0.05	<0.05	<0.05	<0.05
Oleocanthal	n.s.	-----	<0.05	<0.05	<0.05	<0.05	<0.05
HTy	<0.05	<0.05	-----	<0.05	<0.05	<0.05	<0.05
Ty	<0.05	<0.05	<0.05	-----	<0.05	<0.05	<0.05
DHPG	<0.05	<0.05	<0.05	<0.05	-----	<0.05	<0.05
Oleanolic acid	<0.05	<0.05	<0.05	<0.05	<0.05	-----	<0.05
Maslinic acid	<0.05	<0.05	<0.05	<0.05	<0.05	<0.05	-----

HTy: hydroxytyrosol. Ty: tyrosol. DHPG: 3,4-dihydroxyphenylglycol. n.s.: non-significant differences.

**Table 6:** Statistical analysis of the effect of the compounds used on the aortic rings antioxidant markers (oxidized glutathione and percentage of oxidized glutathione with respect to total glutathione) in samples with glucose levels of 100 and 300 mg/dL, as determined using a two-way ANOVA test along with Tukey’s post hoc test.

Glucose 100 mg/dL							
	Oleacein	Oleocanthal	HTy	Ty	DHPG	Oleanolic Acid	Maslinic Acid
Oleacein	-----	<0.05	n.s.	n.s.	<0.05	<0.05	<0.05
Oleocanthal	<0.05	-----	<0.05	<0.05	<0.05	<0.05	<0.05
HTy	<0.05	<0.05	-----	<0.05	<0.05	n.s.	n.s.
Ty	n.s.	<0.05	<0.05	-----	<0.05	<0.05	<0.05
DHPG	<0.05	<0.05	<0.05	<0.05	-----	<0.05	<0.05
Oleanolic acid	<0.05	<0.05	<0.05	<0.05	<0.05	-----	n.s.
Maslinic acid	<0.05	<0.05	n.s.	<0.05	<0.05	n.s.	-----
**Glucose 300 mg/dL**							
	**Oleacein**	**Oleocanthal**	**HTy**	**Ty**	**DHPG**	**Oleanolic Acid**	**Maslinic Acid**
Oleacein	-----	<0.05	<0.05	<0.05	<0.05	<0.05	<0.05
Oleocanthal	<0.05	-----	<0.05	<0.05	<0.05	<0.05	<0.05
HTy	<0.05	<0.05	-----	<0.05	<0.05	n.s.	n.s.
Ty	<0.05	<0.05	<0.05	-----	<0.05	<0.05	<0.05
DHPG	<0.05	<0.05	<0.05	<0.05	-----	<0.05	<0.05
Oleanolic acid	<0.05	<0.05	n.s.	<0.05	<0.05	-----	n.s.
Maslinic acid	<0.05	<0.05	n.s.	<0.05	<0.05	n.s.	-----

HTy: hydroxytyrosol. Ty: tyrosol. DHPG: 3,4-dihydroxyphenylglycol. n.s.: non-significant differences.

## Data Availability

The data presented in this study are available in the article.

## References

[1] Dogné J.M., Hanson J., Praticò D. (2005). Thromboxane, prostacyclin and isoprostanes: Therapeutic targets in atherogenesis. Trends Pharmacol. Sci..

[2] Zhou Y., Khan H., Xiao J., Cheang W.S. (2021). Effects of arachidonic acid metabolites on cardiovascular health and disease. Int. J. Mol. Sci..

[3] Idborg H., Pawelzik S.C. (2022). Prostanoid metabolites as biomarkers in human disease. Metabolites.

[4] He C., Choi H.C., Xie Z. (2010). Enhanced tyrosine nitration of prostacyclin synthase is associated with increased inflammation in atherosclerotic carotid arteries from type 2 diabetic patients. Am. J. Pathol..

[5] Islam K., Islam R., Nguyen I., Malik H., Pirzadah H., Shrestha B., Lentz I.B., Shekoohi S., Kaye A.D. (2025). Diabetes mellitus and associated vascular disease: Pathogenesis, complications, and evolving treatments. Adv. Ther..

[6] Santilli F., Zaccardi F., Liani R., Petrucci G., Simeone P., Pitocco D., Tripaldi R., Rizzi A., Formoso G., Pontecorvi A. (2020). In vivo thromboxane-dependent platelet activation is persistently enhanced in subjects with impaired glucose tolerance. Diabetes Metab. Res. Rev..

[7] An Y., Xu B.T., Wan S.R., Ma X.M., Long Y., Xu Y., Jiang Z.Z. (2023). The role of oxidative stress in diabetes mellitus-induced vascular endothelial dysfunction. Cardiovasc. Diabetol..

[8] Estruch R., Ros E., Salas-Salvadó J., Covas M.I., Corella D., Arós F., Gómez-Gracia E., Ruiz-Gutiérrez V., Fiol M., Lapetra J. (2018). Primary prevention of cardiovascular disease with a Mediterranean diet supplemented with extra-virgin olive oil or nuts. N. Engl. J. Med..

[9] Salvo A., Tuttolomondo A. (2025). The role of olive oil in cardiometabolic risk. Metabolites.

[10] GokulRaj D.K., Jayasuriya R., Ramkumar K.M. (2025). Hydroxytyrosol from Olive Oil Mitigates Endothelial Dysfunction in Diabetic Foot Ulcers via Redox, Inflammatory, and Survival Pathways. J. Nutr..

[11] Achour O., Haffani Y.Z., Mbarek S., Hammami O., Feki M., Zemmel A., Picaud S., Boudhrioua N., Chaouacha-Chekir R.B. (2025). Hydroxytyrosol-Rich Olive Mill Wastewater, a Potential Protector Against Dyslipidemia, Diabetes, and Diabetic Retinopathy in Psammomys obesus. Chem. Biodivers..

[12] Rodriguez-Pérez M.D., Santiago-Corral L., Ortega-Hombrados L., Verdugo C., Arrebola M.M., Martín-Aurioles E., Fernández-Prior M.Á., Bermúdez-Oria A., De La Cruz J.P., González-Correa J.A. (2023). The effect of the extra virgin olive oil minor phenolic compound 3′,4′-dihydroxyphenylglycol in experimental diabetic kidney disease. Nutrients.

[13] Jimenez-Lopez C., Carpena M., Lourenço-Lopes C., Gallardo-Gomez M., Lorenzo J.M., Barba F.J., Prieto M.A., Simal-Gandara J. (2020). Bioactive compounds and quality of extra virgin olive oil. Foods.

[14] Yang J., Liu Z. (2022). Mechanistic pathogenesis of endothelial dysfunction in diabetic nephropathy and retinopathy. Front. Endocrinol..

[15] Brownlee M. (2001). Biochemistry and molecular cell biology of diabetic complications. Nature.

[16] Beauchamp G.K., Keast R.S., Morel D., Lin J., Pika J., Han Q., Lee C.H., Smith A.B., Breslin P.A. (2005). Phytochemistry: Ibuprofen-like activity in extra-virgin olive oil. Nature.

[17] Costa V., Costa M., Videira R.A., Andrade P.B., Paiva-Martins F. (2022). Anti-inflammatory activity of olive oil polyphenols—The role of oleacein and its metabolites. Biomedicines.

[18] Cuffaro D., Bertolini A., Bertini S., Ricci C., Cascone M.G., Danti S., Saba A., Macchia M., Digiacomo M. (2023). Olive mill wastewater as source of polyphenols with nutraceutical properties. Nutrients.

[19] De La Cruz J.P., Osuna-Esteban L., Rodríguez-Pérez M.D., Ortega-Hombrados L., Sánchez-Tévar A.M., Martín-Aurioles E., Fernández-Prior M.Á., Pérez-Burillo S., Espejo-Calvo J.A., González-Correa J.A. (2024). Effect of a triterpenoid-rich olive oil on chronic kidney disease in an experimental model of diabetes mellitus. Nutrients.

[20] Moreno J.J. (2003). Effect of olive oil minor components on oxidative stress and arachidonic acid mobilization and metabolism by macrophages RAW 264.7. Free Radic. Biol. Med..

[21] Yap W.H., Lim Y.M. (2015). Mechanistic perspectives of maslinic acid in targeting inflammation. Biochem. Res. Int..

[22] Giménez-Bastida J.A., González-Sarrías A., Laparra-Llopis J.M., Schneider C., Espín J.C. (2021). Targeting mammalian 5-lipoxygenase by dietary phenolics as an anti-inflammatory mechanism: A systematic review. Int. J. Mol. Sci..

[23] Kumar N., Gorai B., Gupta S., Shiva, Goel N. (2021). Extrapolation of hydroxytyrosol and its analogues as potential anti-inflammatory agents. J. Biomol. Struct. Dyn..

[24] Lama A., Pirozzi C., Mollica M.P., Trinchese G., Di Guida F., Cavaliere G., Calignano A., Mattace Raso G., Berni Canani R., Meli R. (2017). Polyphenol-rich virgin olive oil reduces insulin resistance and liver inflammation and improves mitochondrial dysfunction in high-fat diet fed rats. Mol. Nutr. Food Res..

[25] Iakovis N., Ikonomidis I., Andreadou I., Xanthopoulos A., Chamaidi A., Chrysakis N., Giamouzis G., Skoularigis J., Tseti I., Triposkiadis F. (2023). The Short-Term Effect of Olive Oil Extract Enriched with Hydroxytyrosol on Cardiovascular Function. J. Med. Food.

[26] Patrono C. (2006). The PGH-synthase system and isozyme-selective inhibition. J. Cardiovasc. Pharmacol..

[27] Badimon L., Vilahur G., Rocca B., Patrono C. (2021). The key contribution of platelet and vascular arachidonic acid metabolism to the pathophysiology of atherothrombosis. Cardiovasc. Res..

[28] Cavalca V., Rocca B., Squellerio I., Dragani A., Veglia F., Pagliaccia F., Porro B., Barbieri S.S., Tremoli E., Patrono C. (2014). In vivo prostacyclin biosynthesis and effects of different aspirin regimens in patients with essential thrombocythaemia. Thromb. Haemost..

[29] Di Risola D., Mattioli R., Federico R., Pascarella G., Fontana M., Dainese E., Dufrusine B., Ciogli A., Gasparrini F., Morea V. (2025). Green synthesis and two-step chromatographic separation of thiocanthal and thiocanthol: Two novel biologically active sulfur derivatives of oleocanthal and oleacein from extra virgin olive oil. Food Chem..

[30] Scoditti E., Nestola A., Massaro M., Calabriso N., Storelli C., De Caterina R., Carluccio M.A. (2014). Hydroxytyrosol suppresses MMP-9 and COX-2 activity and expression in activated human monocytes via PKCα and PKCβ1 inhibition. Atherosclerosis.

[31] Léger C.L., Carbonneau M.A., Michel F., Mas E., Monnier L., Cristol J.P., Descomps B. (2005). A thromboxane effect of a hydroxytyrosol-rich olive oil wastewater extract in patients with uncomplicated type I diabetes. Eur. J. Clin. Nutr..

[32] Toniolo A., Buccellati C., Pinna C., Gaion R.M., Sala A., Bolego C. (2013). Cyclooxygenase-1 and prostacyclin production by endothelial cells in the presence of mild oxidative stress. PLoS ONE.

[33] Günther A., Bednarczyk-Cwynar B. (2025). Oleanolic acid: A promising antioxidant—Sources, mechanisms of action, therapeutic potential, and enhancement of bioactivity. Antioxidants.

[34] He Y., Wang Y., Yang K., Jiao J., Zhan H., Yang Y., Lv D., Li W., Ding W. (2022). Maslinic acid: A new compound for the treatment of multiple organ diseases. Molecules.

[35] Kourek C., Makaris E., Benetou V., Magiatis P., Zouganeli V., Dimopoulos S., Georgiopoulos G., Briasoulis A., Paraskevaidis I., Melliou E. (2025). Extra Virgin Olive Oil (EVOO) Improves Vascular Endothelial Function and Hemodynamic Parameters in Patients with Hyperlipidemia: A Post Hoc Analysis of a Randomized Controlled Trial. Nutrients.

[36] Tehrani S.D., Ahmadi A.R., Sadeghi N., Keshani M. (2025). The Effects of the Mediterranean Diet Supplemented with Olive Oils on Pro-Inflammatory Biomarkers and Soluble Adhesion Molecules: A Systematic Review and Meta-Analysis of Randomized Controlled Trials. Nutr. Metab..

[37] Moratilla-Rivera I., Fernández-Millán E., Pérez-Jiménez J., Ramos S., Yanes Ó., Capellades J., Mateos R., Martín M.Á. (2026). Hydroxytyrosol Modulates Arachidonic Acid Metabolism and Purine Catabolism in Individuals with Prediabetes: An Untargeted Metabolomics Study in a Randomized Controlled Trial. Antioxidants.

[38] Davì G., Catalano I., Averna M., Notarbartolo A., Strano A., Ciabattoni G., Patrono C. (1990). Thromboxane biosynthesis and platelet function in type II diabetes mellitus. N. Engl. J. Med..

[39] De La Cruz Cortés J.P., Pérez de Algaba I., Martín-Aurioles E., Arrebola M.M., Ortega-Hombrados L., Rodríguez-Pérez M.D., Fernández-Prior M.Á., Bermúdez-Oria A., Verdugo C., González-Correa J.A. (2021). Extra virgin olive oil polyphenols improve the protective effects of hydroxytyrosol in an in vitro model of hypoxia-reoxygenation of rat brain. Brain Sci..

